# Supplementary material for: Comparative Metabolomics Profiling Reveals Key Metabolites and Associated Pathways Regulating Tuber Dormancy in White Yam (Dioscorea rotundata Poir.)
Source: Metabolites. 2023 Apr 28;13(5):610. doi: 10.3390/metabo13050610 (PMC10223290; doi:10.3390/metabo13050610)
Supplement: Supplementary file 1 [file metabolites-13-00610-s001.zip › Figure S1a.pdf]

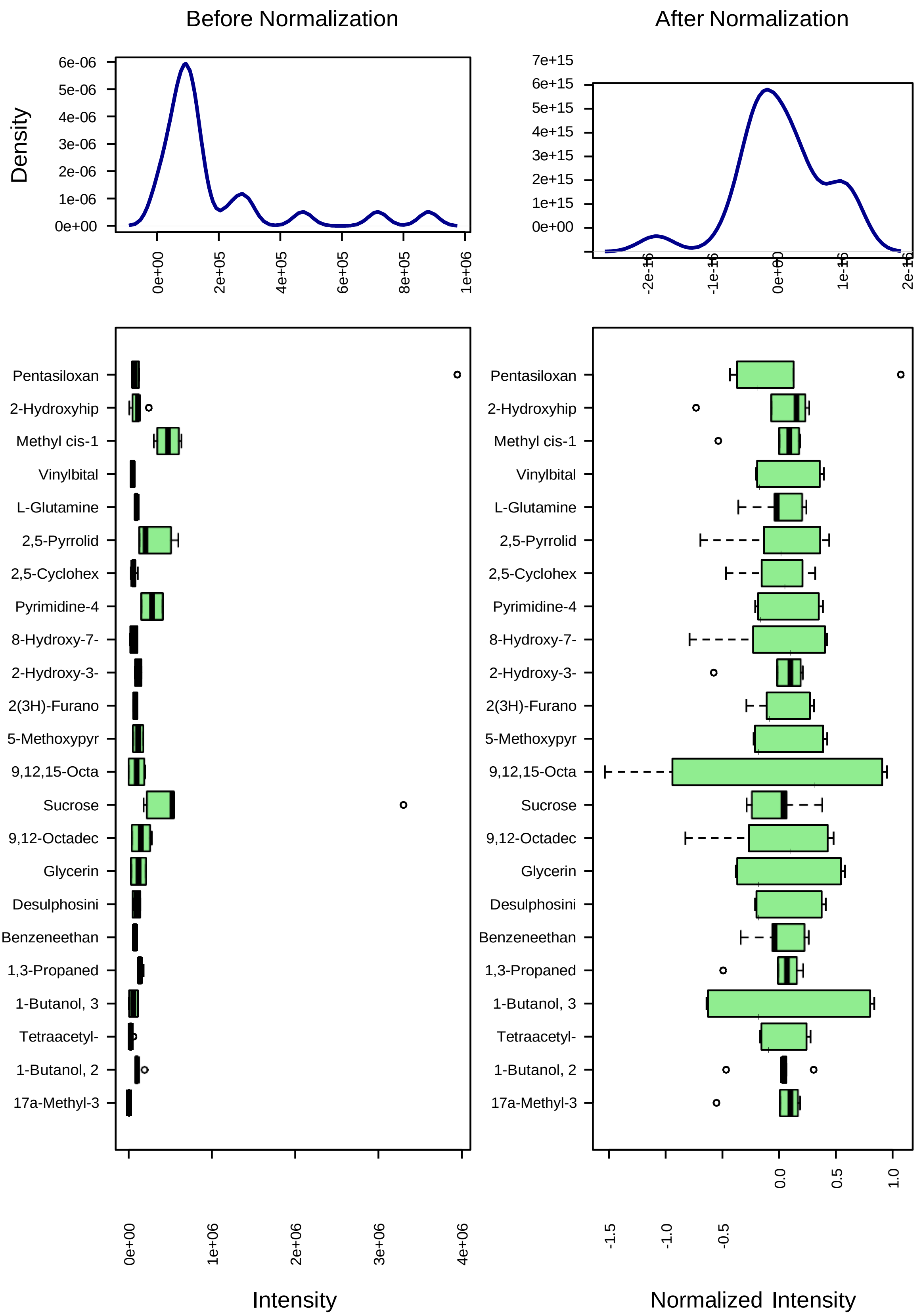

Figure S1: Box plots and kernel density plots before and after normalization. The boxplots show at most 50 features due to space limit. The density plots are based on all samples. Selected methods : Row-wise normalization: Normalization to constant sum; Data transformation: Log10 Normalization; Data scaling: Mean Centering.
