# Supplementary material for: Comparative Metabolomics Profiling Reveals Key Metabolites and Associated Pathways Regulating Tuber Dormancy in White Yam (Dioscorea rotundata Poir.)
Source: Metabolites. 2023 Apr 28;13(5):610. doi: 10.3390/metabo13050610 (PMC10223290; doi:10.3390/metabo13050610)
Supplement: Supplementary file 1 [file metabolites-13-00610-s001.zip › Figure S1b.pdf]

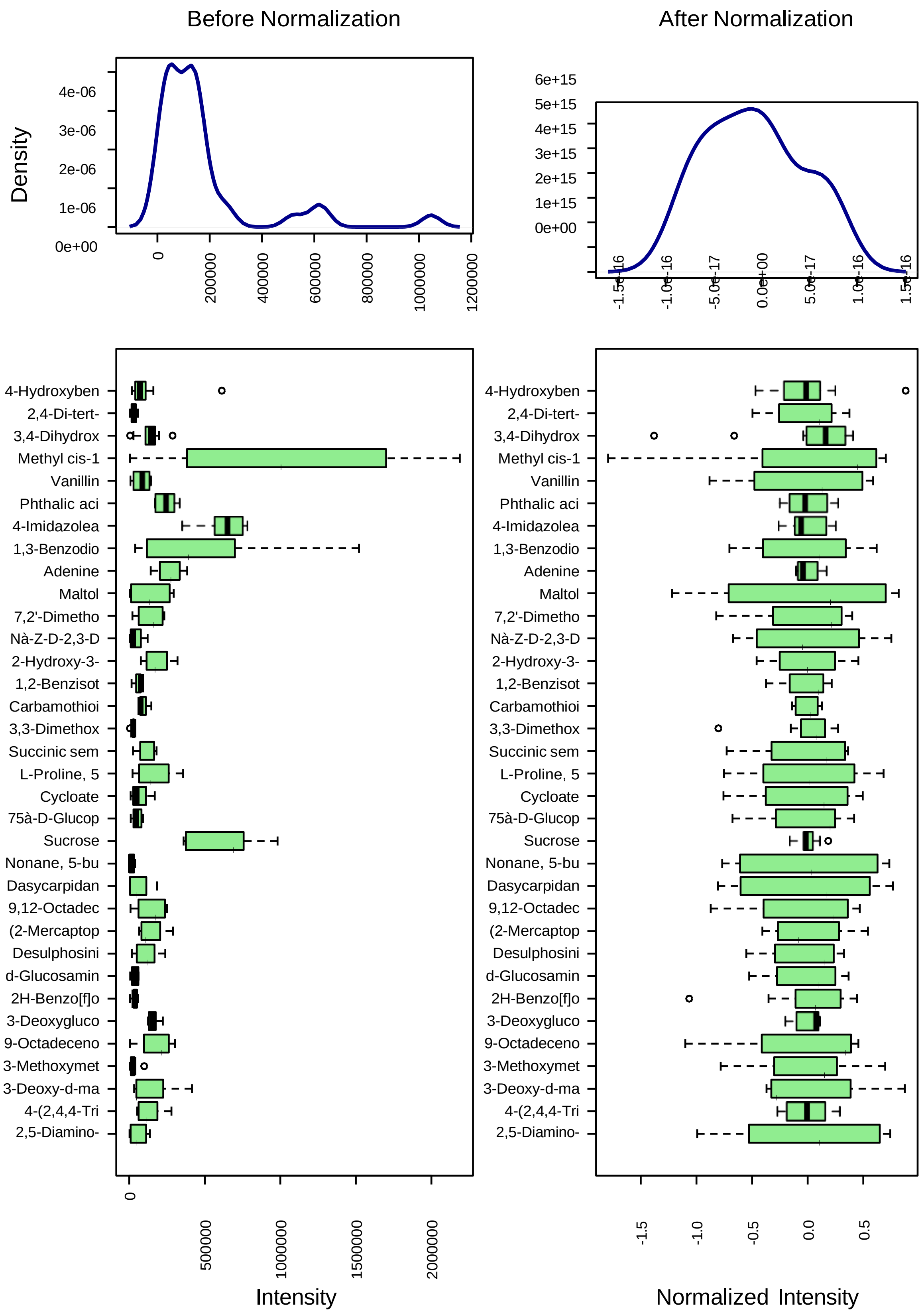

Figure S1: Box plots and kernel density plots before and after normalization for *Obiaoturugo*. The boxplots show at most 50 features due to space limit. The density plots are based on all samples. Selected methods : Row- wise normalization: Normalization to constant sum; Data transformation: Log10 Normalization; Data scaling: Mean Centering.
