# Supplementary material for: Comparative Metabolomics Profiling Reveals Key Metabolites and Associated Pathways Regulating Tuber Dormancy in White Yam (Dioscorea rotundata Poir.)
Source: Metabolites. 2023 Apr 28;13(5):610. doi: 10.3390/metabo13050610 (PMC10223290; doi:10.3390/metabo13050610)
Supplement: Supplementary file 1 [file metabolites-13-00610-s001.zip › Table S1.pdf]

	PC1	PC2	PC3	PC4	PC5	PC6	PC7	PC8
42	-0.18202	-0.1044	0.34439	0.024943	-0.25412	-0.2098	0.001593	0.18476
43	-0.10765	0.014159	0.050064	0.092122	-0.05121	0.070356	-0.01756	0.015367
44	-0.00649	0.11442	0.087351	-0.03789	-0.276	-0.06511	0.018598	0.038764
55	0.028953	0.28897	0.38414	-0.17562	0.16488	-0.00473	0.24693	-0.11351
57	-0.08894	0.042124	0.036068	-0.07746	-0.05865	0.10614	0.16845	0.00764
58	0.041758	0.40145	-0.18964	-0.00501	0.22413	-0.31517	0.1644	0.65183
59	-0.07184	0.13295	-0.3317	-0.03874	-0.31389	0.19915	0.16489	0.033132
60	0.15929	0.013197	0.11989	0.38079	-0.07539	0.15679	-0.02402	0.16023
61	0.13024	0.11992	0.066899	-0.13987	-0.03145	0.16305	0.24124	-0.00512
67	-0.21496	-0.08751	-0.08419	0.2531	0.086184	0.16882	0.006342	-0.21078
69	-0.15861	0.28885	0.038933	0.07656	-0.07661	0.027537	0.10615	-0.0359
71.025	0.065493	0.32512	-0.0008	0.36068	-0.16458	0.12965	-0.0091	-0.32293
73	0.0889	-0.05861	-0.02102	-0.0007	0.069543	-0.07468	-0.15613	0.015419
79	-0.5418	-0.13223	-0.17872	0.19092	0.011103	0.061277	0.16169	0.29532
82	0.058758	0.13509	0.18616	0.25342	-0.09473	0.054514	0.004511	0.089384
83	-0.19924	0.10912	0.14522	0.16755	-0.00997	-0.00379	-0.00302	0.010324
84	0.25161	-0.0147	-0.01476	0.085226	-0.32906	0.17736	0.067225	0.16711
85	-0.37582	0.31857	-0.22943	-0.07619	0.12053	0.13969	0.25228	-0.2507
98	-0.14847	0.061183	0.32674	0.018502	-0.04981	-0.09443	-0.01088	0.010041
100	0.034538	0.16962	0.17035	0.29967	-0.19113	0.017224	-0.0414	0.10912
104	0.020198	-0.0705	0.23144	-0.14078	0.27733	0.15222	0.21392	-0.04334
108	-0.04187	0.17687	0.32305	-0.13386	0.013998	-0.04112	0.14	-0.05026
122	-0.15411	-0.13707	0.2196	0.009888	0.066694	0.022618	0.009515	0.025794
126	0.12969	0.045139	-0.07498	-0.18778	-0.31297	0.15	0.18268	0.061616
133	0.077455	-0.09081	0.10912	0.23107	0.24208	0.24399	0.099415	0.063637
135	-0.17298	-0.12062	0.044715	-0.27719	-0.34184	-0.04014	0.033342	0.038633
138.05	0.1947	-0.25206	-0.04019	0.069153	0.11692	0.21451	0.44048	0.12155
139	-0.05502	-0.16674	0.071659	-0.18738	-0.26968	-0.00053	0.17884	-0.0527
149	-0.32051	-0.19654	0.081825	0.086653	-0.04744	-0.00404	-0.08708	0.051514
155.05	0.089827	-0.14463	0.010357	0.025742	-0.03173	0.19587	0.10416	0.09191
207.05	0.09527	-0.24767	-0.06522	0.31962	-0.06173	-0.57803	0.55225	-0.24795
281.05	0.098775	0.15148	-0.16943	0.031785	-0.07683	-0.31505	-0.07646	-0.21594

PC9	PC10	PC11	PC12	PC13	PC14	PC15	PC16	PC17
-0.10552	-0.41891	-0.40199	-0.17946	-0.1837	0.23908	0.12977	0.13808	0.31446
-0.01408	-0.23072	0.26163	-0.18029	0.090844	0.19563	-0.80783	0.31126	-0.00402
-0.03281	-0.09331	-0.03498	-0.03875	-0.17081	-0.04537	0.028059	0.008302	-0.0307
-0.01085	0.10723	0.000829	-0.04202	0.08638	0.088262	-0.04551	-0.03424	-0.08453
0.28263	0.059298	-0.23923	-0.07202	0.14006	-0.67087	-0.23916	0.068999	0.17883
-0.07645	0.19951	0.078041	-0.17063	-0.23594	-0.01311	-0.01891	0.092464	0.049988
0.12046	-0.05996	-0.12742	0.058052	0.12866	0.19283	0.03339	0.045665	-0.13931
0.018471	-0.10402	0.17892	0.066142	0.064866	-0.07621	0.13981	0.062285	0.01347
0.087387	-0.02209	0.060836	-0.10799	0.080812	0.10462	0.090214	0.098777	-0.00773
-0.09894	0.086574	0.2479	-0.38249	-0.54757	-0.12602	0.048646	0.04409	-0.15509
-0.01027	-0.03748	-0.07388	0.2088	0.1055	0.069301	-0.06992	-0.12866	-0.09414
-0.10943	0.46426	-0.40575	-0.20857	-0.05498	0.13183	-0.03176	0.14486	0.1852
-0.03605	0.022747	-0.01645	0.075696	0.087691	-0.34675	0.063878	0.465	0.34004
-0.03078	0.21262	-0.02368	-0.05779	0.37894	0.081885	0.069511	-0.0222	0.043414
-0.01748	-0.06135	0.13706	0.11194	0.034665	-0.0996	0.042317	-0.05003	-0.02506
-0.02763	-0.00594	-0.01153	0.13492	0.082092	-0.02655	-0.00849	-0.09572	-0.08788
0.092163	-0.06027	0.19956	-0.10924	-0.01196	-0.0836	0.15168	0.11152	-0.03755
-0.04306	-0.42072	0.16249	0.15162	-0.17805	-0.12306	0.19418	0.053946	0.26735
-0.04453	0.062754	0.10184	-0.00157	-0.02069	-0.17024	-0.00713	-0.11682	-0.08823
-0.03663	-0.0655	0.14221	0.17019	0.006538	-0.15286	0.007517	-0.10784	-0.0538
0.077425	0.032469	0.052177	-0.22507	0.13081	0.12897	0.16449	0.17835	0.033702
-0.01524	0.049241	0.088011	-0.03504	0.006919	-0.06521	-0.01514	-0.08003	-0.04479
0.021681	0.062767	0.059621	-0.13191	0.05246	-0.05445	0.10888	0.046942	-0.06633
0.11784	-0.00311	0.080144	-0.16096	0.007972	0.022916	0.094438	0.092718	-0.08331
0.069427	-0.07372	0.065032	-0.06104	0.18536	0.13456	0.20916	0.20193	0.05831
0.066782	0.12611	0.048676	-0.22954	-0.06798	-0.13322	0.048018	-0.00986	-0.18913
-0.54093	-0.16607	-0.3466	0.026602	-0.06612	-0.21259	-0.15127	-0.10234	-0.24024
-0.49598	0.35527	0.32898	0.33694	-0.00943	0.075514	-0.0032	0.18686	0.28455
0.018464	0.071015	-0.02282	-0.0655	0.060233	-0.06414	0.084591	0.004157	-0.14469
0.11106	0.055276	0.13163	-0.18986	-0.05091	0.065363	-0.14202	-0.62217	0.59041
0.31008	0.020382	0.078276	0.081493	-0.03301	0.037143	-0.0223	0.040641	-0.00759
-0.40388	-0.19735	0.16115	-0.47792	0.49247	-0.13979	0.12915	-0.14581	0.018264

PC18
0.1649
-0.04003
-0.58095
0.18603
0.076315
0.099968
0.46452
-0.09722
-0.16493
0.17004
0.0435
-0.10082
0.10178
-0.29911
-0.00442
0.21959
0.037895
-0.0989
-0.08782
0.15794
-0.0121
-0.04617
0.066356
-0.17417
0.10699
0.16603
-0.03306
0.10222
0.008442
0.073877
0.000871
0.069522
