# Supplementary material for: Comparative Metabolomics Profiling Reveals Key Metabolites and Associated Pathways Regulating Tuber Dormancy in White Yam (Dioscorea rotundata Poir.)
Source: Metabolites. 2023 Apr 28;13(5):610. doi: 10.3390/metabo13050610 (PMC10223290; doi:10.3390/metabo13050610)
Supplement: Supplementary file 1 [file metabolites-13-00610-s001.zip › Table S2.pdf]

M/z	PC1	PC2	PC3	PC4	PC5	PC6	PC7	PC8
42	-0.19535	0.36057	-0.11035	0.028485	-0.13389	-0.34207	0.15151	0.40744
43	-0.06053	-0.02949	0.12399	0.081631	0.007596	0.022204	0.2369	-0.05611
44	0.2576	-0.06833	-0.03679	-0.03634	-0.07119	-0.06763	0.30658	0.035942
45	0.046287	-0.06551	0.52693	0.068034	-0.02401	-0.11988	0.083192	-0.10945
55	-0.33391	0.063941	0.077833	0.065665	-0.0212	0.087452	0.007764	-0.19735
57	0.032958	0.052198	0.022189	0.03651	0.002128	-0.06256	0.009346	-0.17313
58	0.15596	0.11979	-0.01118	-0.13871	-0.48845	0.12856	0.39001	-0.05592
59	-0.15493	0.032863	0.16204	0.11805	-0.04887	0.06781	0.032804	-0.11579
60	-0.17299	-0.00626	-0.00901	0.1325	-0.00871	0.11463	0.055223	-0.11209
61	0.17694	0.05315	0.018283	0.053584	-0.05315	-0.01199	0.013171	0.042235
67	-0.26223	0.046967	0.16301	0.12017	-0.04157	0.085103	0.013155	-0.16771
69	-0.22414	-0.17982	-0.34958	0.069684	-0.0546	0.32044	0.24287	-0.16852
71	0.020865	0.34894	0.18269	-0.11028	0.21189	0.7402	0.039897	0.28894
73	-0.0098	0.000564	-0.01419	-0.16947	0.03741	-0.09552	0.15953	-0.15387
75	0.061295	-0.18127	-0.26361	-0.04498	-0.0178	0.039767	0.050798	-0.15898
83	0.055136	-0.22046	0.33934	0.23055	-0.05784	0.060041	0.10839	0.077255
84	0.18857	-0.10922	-0.35641	-0.11647	0.014632	0.02508	0.058549	0.1506
85	0.14527	0.21973	0.025373	0.042675	-0.07071	-0.03562	-0.03828	-0.04575
89.05	0.064532	-0.02363	-0.16711	0.44353	-0.04272	0.21461	-0.05847	0.3082
100	0.01802	-0.05744	0.026601	0.063062	-0.03168	0.046577	0.053544	0.015737
102	-0.10743	0.054502	0.025439	0.050426	-0.03599	0.055867	0.022368	-0.09138
103	0.15272	0.089881	-0.02033	0.030343	-0.03186	0.000321	-0.00804	0.054061
104	-0.18373	0.2181	-0.23578	-0.09276	-0.0143	0.050382	-0.0306	-0.17582
122	0.095083	0.29189	-0.03558	0.005732	-0.06737	-0.03545	-0.06029	-0.09534
126	0.004305	0.53266	-0.04947	-0.0229	-0.08661	-0.05818	-0.13352	-0.24139
135	-0.02455	0.04421	-0.05357	0.01446	-0.02955	0.039906	0.021681	-0.04152
138.05	-0.23092	-0.05935	-0.02899	-0.18714	0.18031	-0.04124	-0.45854	0.12977
139	0.074877	0.019209	0.046833	0.11701	-0.11004	0.054896	-0.03198	0.194
149	0.08478	0.025814	-0.1071	-0.01662	-0.01921	0.026001	0.074858	0.047745
151	0.22171	0.242	0.10588	0.082067	-0.08893	-0.06019	-0.04669	-0.02677
155.05	-0.4921	0.041031	-0.0124	0.10766	-0.0926	-0.21299	0.2748	0.38359
207.05	0.13933	0.1864	-0.10543	0.34816	0.72238	-0.16559	0.33315	-0.09771
263.15	-0.14631	0.064594	-0.06501	0.021958	0.026522	0.039918	0.17955	-0.24649
281.05	-0.05925	2.20E-05	0.17982	-0.62773	0.26437	-0.00585	0.30084	0.129

PC9	PC10	PC11	PC12
-0.12561	0.14443	-0.04225	0.15314
0.43916	0.061643	-0.46567	-0.09973
0.099211	-0.23644	0.19297	-0.56771
-0.2124	0.14688	-0.16177	-0.2898
0.19501	-0.21366	0.29608	-0.29501
-0.03583	0.37699	0.38725	-0.10146
0.082961	-0.24364	-0.16721	0.16977
0.024681	-0.03623	-0.01926	0.27179
0.062858	0.048975	0.11269	0.12127
-0.07593	0.090503	0.10364	-0.1381
0.071986	-0.14331	0.17247	0.08624
-0.68214	0.019265	-0.16039	-0.13792
0.032148	0.14787	-0.044	-0.12836
-0.0375	0.15274	-0.119	-0.06682
0.22177	0.16577	-0.13696	-0.0592
-0.1031	0.058256	0.080942	0.020172
0.10243	0.13507	0.20885	-0.1207
-0.09334	0.059779	0.051077	0.093291
0.17489	0.15644	0.07908	0.002012
0.01513	0.044757	0.079761	0.036391
0.055019	-0.01339	0.012612	-0.06236
-0.03667	-0.02006	-0.36187	-0.05396
0.17029	-0.04423	-0.00947	-0.04413
-0.05717	0.036982	0.02728	-0.10703
-0.05112	-0.02233	-0.05892	-0.21342
0.050457	0.022527	0.054161	0.050641
-0.0699	-0.21758	-0.22399	-0.24216
-0.13844	-0.58164	0.13967	0.064572
0.009913	-0.19182	0.082305	0.049833
-0.16667	0.080314	0.056461	-0.01301
0.017409	0.086919	0.003	-0.24789
-0.03613	-0.21518	-0.06751	0.063134
0.008778	0.076682	0.092299	0.16109
-0.0412	-0.0037	0.20438	0.15956
