# Supplementary figures and images for: Comparative Metabolomics Profiling Reveals Key Metabolites and Associated Pathways Regulating Tuber Dormancy in White Yam (Dioscorea rotundata Poir.)
Source: Metabolites. 2023 Apr 28;13(5):610. doi: 10.3390/metabo13050610 (PMC10223290; doi:10.3390/metabo13050610)

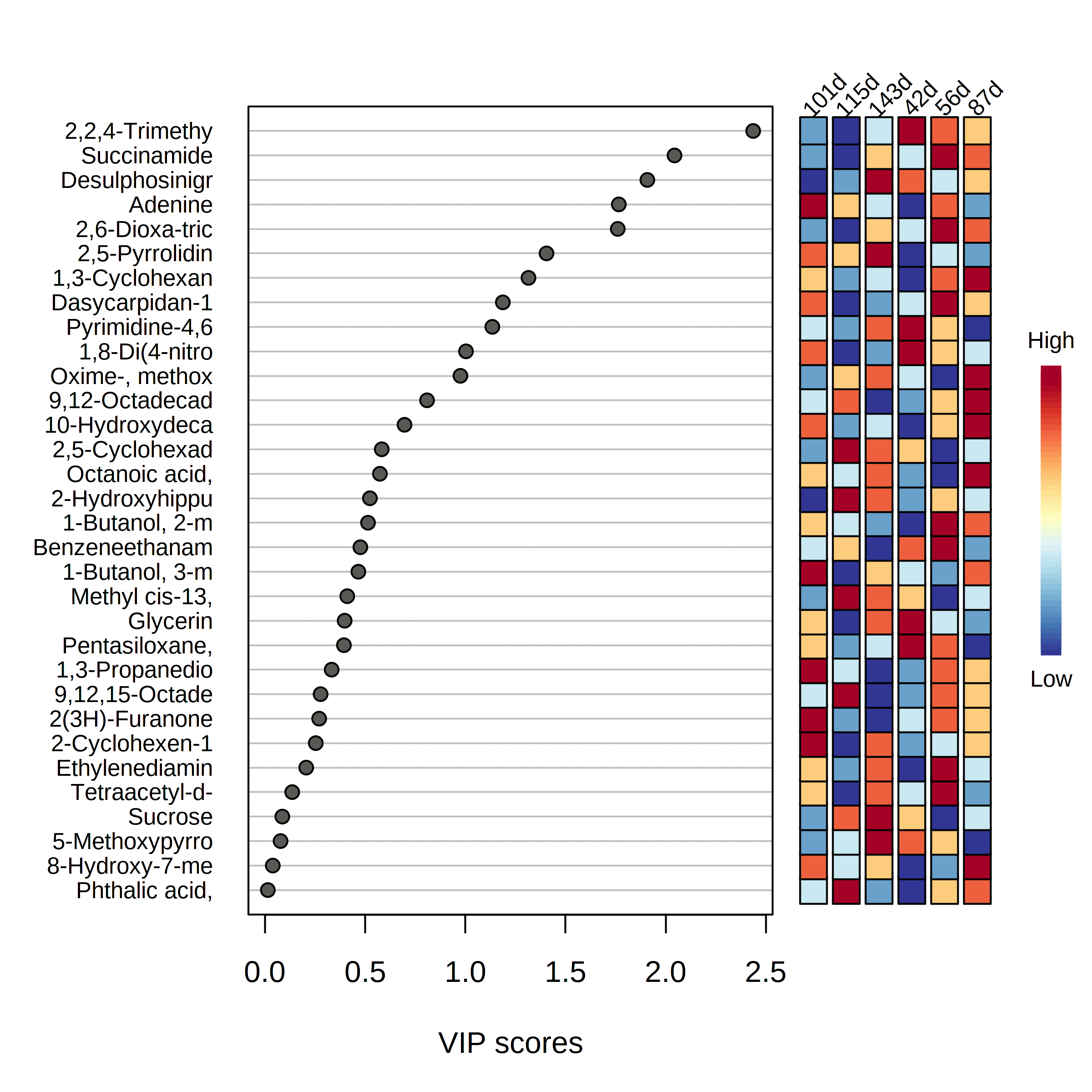

Supplement: Supplementary file 1 [file metabolites-13-00610-s001.zip › Figure S2.png]

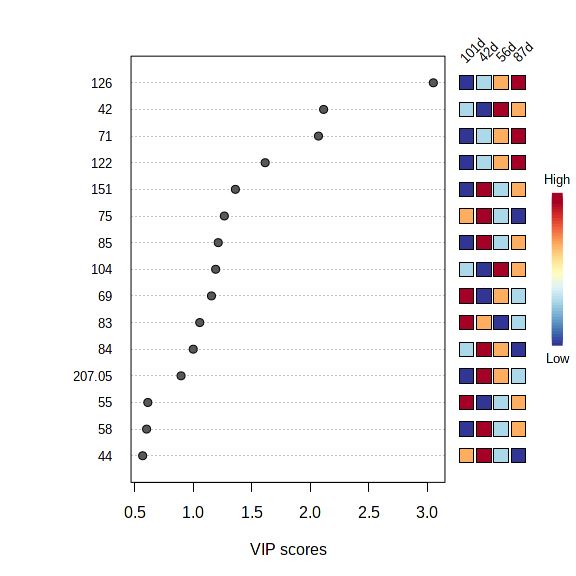

Supplement: Supplementary file 1 [file metabolites-13-00610-s001.zip › Figure S3.png]
